# Clinical and molecular findings in a Moroccan patient with popliteal pterygium syndrome: a case report

**DOI:** 10.1186/1752-1947-8-471

**Published:** 2014-12-29

**Authors:** Ilham Ratbi, Nawfal Fejjal, Marie Legendre, Nathalie Collot, Serge Amselem, Abdelaziz Sefiani

**Affiliations:** 8 Centre de génomique humaine, Faculté de médecine et pharmacie, Université Mohammed V Souissi, Angle Avenue Allal El Fassi et Mfadel Cherkaoui, 10100 Rabat, Morocco; 9 Service de chirurgie plastique pédiatrique, Hôpital des Enfants, Boulevard Ibn Rochd, 10100 Rabat, Morocco; 10 U.F. de Génétique moléculaire, Hôpital Trousseau, APHP, 26, Avenue du Docteur Arnold-Netter, 75012 Paris, France; 11 Département de Génétique médicale, Institut National d’Hygiène, 27, Avenue Ibn Batouta, 11400 Rabat, Morocco

**Keywords:** Popliteal pterygium syndrome, Autosomal dominant, *IRF6* gene

## Abstract

**Introduction:**

Popliteal pterygium syndrome is a congenital malformation that includes orofacial, musculoskeletal and genitourinary anomalies. It is a rare autosomal dominant disorder due to a mutation of the *IRF6* gene on 1q32.2.

**Case presentation:**

A one-month-old Moroccan baby boy was diagnosed with typical features of popliteal pterygium syndrome and carried the c.250C>T; p.Arg84Cys mutation of the *IRF6* gene.

**Conclusions:**

We report on the first description of a Moroccan popliteal pterygium syndrome patient. This diagnosis allowed us to provide an appropriate course of management to the patient and offer genetic counseling to his family.

## Introduction

Popliteal pterygium syndrome (PPS, OMIM:19500) is a rare autosomal dominant malformative disorder, characterized by orofacial, cutaneous, musculoskeletal, and genital anomalies [1]. There is considerable phenotypic variability within and between families [2]. An autosomal recessive form of PPS, described as lethal-type popliteal pterygium syndrome (LPPS, MIM 263650) and also known as Bartsocas-Papas syndrome, is also described [3]. The incidence of PPS is approximately 1 in 300,000 live births [4]. Major anomalies in PPS are cleft lip and/or palate, lower lip pits or sinuses, popliteal webbing, syndactylies, and a distinctive nail anomaly comprising a pyramidal skin fold extending from the base to the top of the nails [1, 4]. Male patients may have bifid scrotum and cryptorchidism; hypoplastic labia majora are observed in females. Other clinical findings include oral adhesions, syngnathia, ankyloblepharon, talipes, spina bifida occulta, bifid ribs, and short sternum. There is no growth delay and intelligence is usually normal [1]. The interferon regulatory factor-6 gene (*IRF6*) on 1q32.2 was identified by Kondo *et al*. as responsible for both PPS and Van der Woude syndrome (VWS), a more common oral cleft syndrome (VWS, MIM 119300) [5].

We report on the first description of a Moroccan patient with popliteal pterygium syndrome carrying a missense mutation of the hotspot arginine 84.

## Case presentation

**Figure 1:**
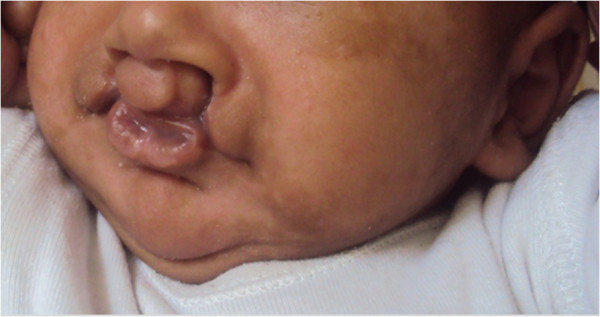
**Index case showing a bilateral cleft lip with two large pits on his lower lip and achromic spots in his face.**

**Figure 2:**
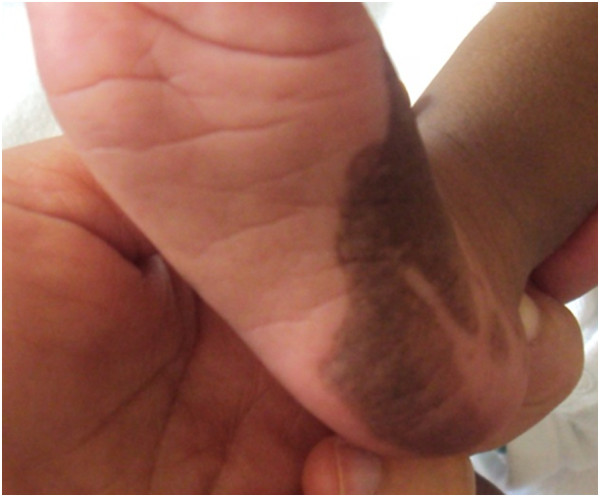
**Verrucous hamartoma on the sole of our patient’s feet.**

**Figure 3:**
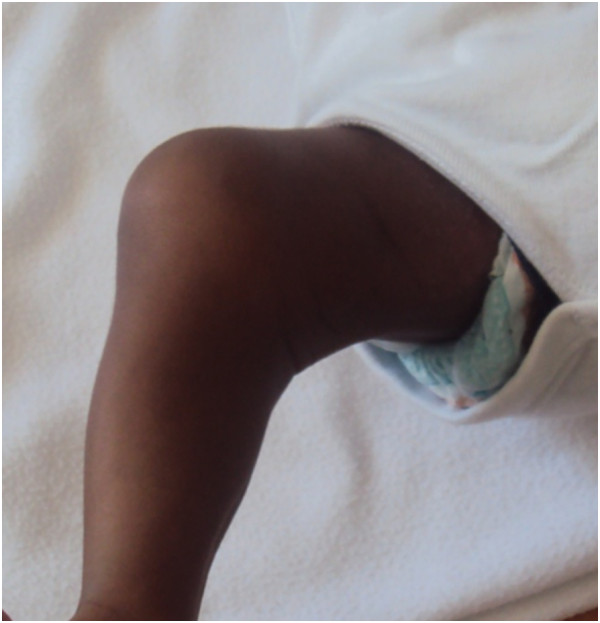
**Right popliteal pterygium.**

**Figure 4:**
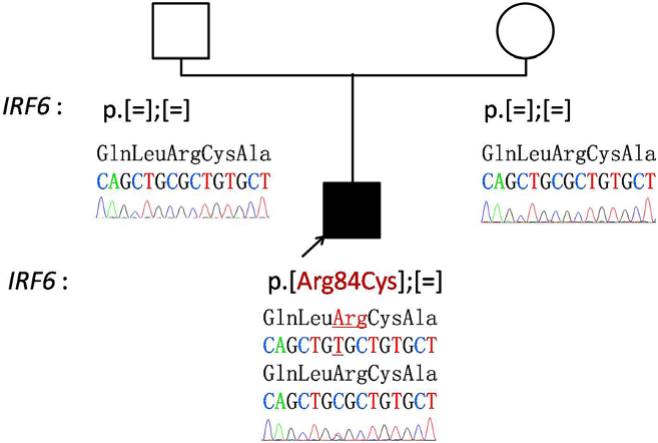
**Pedigree and electrophoregrams showing a normal sequence (for the two parents) and the heterozygous C>T substitution of the interferon regulatory factor-6 (** ***IRF6*** **) gene causing the p.Arg84Cys amino acid change in the interferon regulatory factor 6 protein (for our patient).**

A one-month-old Moroccan baby boy was referred to our institute for a medical genetic consultation with a chief complaint of malformations diagnosed as popliteal pterygium syndrome. He was the fourth liveborn child of a healthy nonconsanguineous couple, aged 35 for the mother and 49 for the father, with no particular familial history. On clinical examination, both the parents and other siblings were normal. During pregnancy, the mother had no history of drug ingestion, abdominal trauma, or radiographic examination. The pregnancy was not medically followed, but it was reported without complications. The baby was born at term by vaginal delivery. His Apgar score was good. At birth, he weighed 3,500g; his length was 52cm; his head circumference was 36cm. On general physical examination, our patient had a bilateral cleft lip and palate with two large pits on the lower lip and oral synechias that did not restrict feeding (Figure 1). He presented several achromic spots of different shapes and sizes on his face. They were irregular but with a well-defined border. On Wood lamp examination, they appeared off-white. There was an asymmetrical large-size pigmented nevus on the sole of his right foot, without relevant nails anomalies (Figures 1 and 2). He also had right popliteal pterygium and bilateral syndactyly of four to five toes (Figure 3). The urogenital examination showed a phimosis. X-rays of the skull, transfontanellar, abdomino-genitourinary and cardiac ultrasonography were normal. The results of all laboratory examinations were within normal limits. Informed consent was obtained from the proband’s parents prior to molecular analysis. Peripheral blood was collected from the affected child and his parents. Molecular genetic testing for suspected PPS was performed by Sanger sequencing of the entire coding region and flanking introns of the *IRF6* gene (NM_006147.3). This led to the identification of the heterozygous c.250C>T; p.Arg84Cys mutation in exon 4 (Figure 4). This mutation occurred *de novo*, as it was not revealed in the hematologic cells of the parents.

Our patient was scheduled for reconstructive surgery of his cleft lip and palate, oral synechiae and popliteal pterygium.

## Discussion

Popliteal pterygium syndrome (PPS) is an oral cleft syndrome with additional clinical features including webbed skin of the legs, genital hypoplasia and/or oral synechiae [1]. PPS and VWS are allelic caused by mutations of the *IRF6* gene on 1q32.2 [2]. It encodes a transcription factor containing a helix-loop-helix (HLH) domain for DNA binding and a Smad interferon regulatory factor-binding domain (SMIR) for protein binding. IRF6 is involved in epithelial differentiation as a component of a regulatory feedback loop that controls the proliferative potential of epidermal cells [6]. The patient described here presented cutaneous lesions. IRF6 is also a potential tumor suppressor gene in squamous cell carcinomas acting on a gene network that contributes to the regulation of cancer cell invasiveness and proliferation [6]. *IRF6* missense mutations are found almost exclusively in the HLH DNA-binding domain or the protein-binding/SMIR domain [2, 7]. Amino acids that are mutated in PPS are essential for the DNA binding in particular the arginine at position 84, which is the only one able to constitute a hydrogen bond with the guanine of the target DNA sequence. Two major dominant negative mutations p.Arg84Cys and p.Arg84His are frequently reported for PPS [3, 8]. In the patient described here, we screened first for PPS because of the typical facial features and the pterygium poplitea. He was heterozygous for a *de novo* p.Arg84Cys mutation, previously reported in patients with PPS and considered deleterious [5, 7, 9]. It was also reported in a patient with VWS and in an asymptomatic individual, supporting the variability in the clinical expression and the incomplete penetrance of the disease phenotype in this syndrome [8].

## Conclusions

In conclusion, we report on the first clinical and molecular description of a Moroccan patient with PPS syndrome. This diagnosis allowed us to provide an appropriate course of management to the patient, and offer genetic counseling to his family.

## Consent

Written informed consent was obtained from the patient’s legal guardian(s) for publication of this case report and any accompanying images. A copy of the written consent is available for review by the Editor-in-Chief of this journal.

## Abbreviations

HLH: helix-loop-helix
IRF6: interferon regulatory factor 6
LPPS: lethal-type popliteal pterygium syndrome
PPS: pterygium popliteal syndrome
SMIR: Smad interferon regulatory factor-binding domain
VWS: Van der Woude syndrome.
